# A Novel Cuproptosis-Related Gene Signature Predicts Prognosis in Papillary Thyroid Carcinoma Patients

**DOI:** 10.2174/0113862073333880240819105537

**Published:** 2024-08-27

**Authors:** Jun Cao, Shijia Zhang, Kehui Zhou, Xiaochun Mao, Ming Zhao, Jinbiao Shang, Xiabin Lan

**Affiliations:** 1 Department of Head and Neck and Rare Oncology, Zhejiang Cancer Hospital, Hangzhou Institute of Medicine (HIM), Chinese Academy of Sciences, Hangzhou, Zhejiang 310022, China;; 2 Postgraduate Training Base Alliance of Wenzhou Medical University (Zhejiang Cancer Hospital), Hangzhou, Zhejiang, 310022, China;; 3 Department of Thyroid Surgery, Zhejiang Cancer Hospital, Hangzhou Institute of Medicine (HIM), Chinese Academy of Sciences, Hangzhou, Zhejiang 310022, China;; 4 Key Laboratory of Head & Neck Cancer Translational Research of Zhejiang Province, Hangzhou, Zhejiang 310022, China

**Keywords:** Thyroid cancer, cuproptosis, gene signature, prognosis, TCGA, quantitative real-time polymerase chain reaction (qRT-PCR)

## Abstract

**Background:**

Cuproptosis, a novel form of cell death mediated by protein lipoylation, is intricately linked to mitochondrial metabolism. However, the clinical association of cuproptosis-related genes (CRGs) in thyroid cancer remains unclear. In this study, we performed a systematic investigation on the differential expression and genetic alterations of CRGs in papillary thyroid carcinoma (PTC) and constructed a CRG signature to predict the prognosis of PTC patients.

**Methods:**

We integrated the data of The Cancer Genome Atlas (TCGA) database and analyzed the expression of 10 CRGs in PTC. CRG signature was constructed using univariate Cox regression and the least absolute shrinkage and selection operator (LASSO) Cox regression. In addition, the signature-related molecular features were validated by a combination of functional enrichment, Cox regression, and immune infiltration analysis. Independent validation cohort and quantitative real-time polymerase chain reaction (qRT-PCR) were used to validate the expression of differentially expressed CRG (CDKN2A).

**Results:**

Thyroid cancer patients could be divided into two subtypes (high and low CRG score groups). We found that the overall survival (OS) of patients was lower in the high CRG score group (HCSG) than in the low CRG score group (LCSG) (*P <* 0.001). The area under the curve (AUC) values for 3 years, 5 years, and 8 years were 0.872, 0.941, and 0.976, respectively. Cox regression analysis indicated that the CRG score could serve as an independent prognostic indicator for PTC. Functional enrichment analysis indicated that the CRG prognostic signature was also associated with the tumor immune microenvironment. In HCSG, the immune suppression cell score was significantly higher than in LCSG. In addition, we identified the expression of CRG (CDKN2A) by qRT-PCR, and the results aligned with the TCGA database.

**Conclusion:**

Our CRG signature demonstrates excellent predictive capabilities for the prognosis of PTC patients. CRGs may play an important role in tumorigenesis and could be used to predict the immunotherapy efficacy of PTC.

## 1 INTRODUCTION

Thyroid Cancer (TC), particularly thyroid papillary carcinoma (PTC), is the most common type of malignant endocrine tumor, and its global incidence has experienced a significant rise over the past few decades [1, 2]. Accounting for more than 80% of all thyroid cancer cases, PTC poses a major public health concern due to its increasing prevalence [3-5]. Most PTCs are indolent and have a good prognosis, with a 10-year survival rate of >90% [6]. However, clinicopathological features, such as age, tumor diameter, extrathyroidal invasion, cervical lymph node metastasis, and distant organ metastasis, are considered to be important factors affecting prognosis, leading to a high recurrence rate [4, 7]. It is reported that approximately 1.7%-15% of PTC patients experience distant metastases [8-11], which significantly impair their survival rates. Tumor recurrence and metastasis obstruct the clinical treatment and survival of patients. So far, the molecular mechanisms of PTC recurrence and metastasis are not fully understood; therefore, it is important to explore new therapeutic targets and construct a signature that can predict the prognosis of PTC and guide clinical treatment.

Copper, as a redox-active element, has many important physiological functions in the maintenance of thyroid activity and lipid metabolism. Copper is not only involved in the production of hemoglobin, myelin, and melanin, but it is also essential for thyroid function by stimulating the production of thyroid hormones (T4) and preventing excessive absorption of T4 by controlling calcium levels [12, 13]. High concentrations of copper can induce tumor growth, proliferation, and carcinogenesis by damaging deoxyribonucleic acid (DNA) through toxic free radicals [14]. Some studies have reported elevated copper levels in various malignant tumors compared to normal tissues. Copper accumulation is associated with cancer growth, angiogenesis, and metastasis [15-17]. Research indicated that the serum copper levels in TC patients are elevated, suggesting that there might be a relationship between changes in copper levels and the development of TC [12, 13]. Tsvetkov *et al*. introduced a novel cell death mechanism termed cuproptosis. Different from all other known cellular regulation death mechanisms (including apoptosis, ferroptosis, pyroptosis, and necrosis), cuproptosis is a mitochondrial stress. Copper directly binds to lipoylated components of the tricarboxylic acid (TCA) cycle, leading to the aggregation of lipoylated proteins and, subsequently, the loss of iron-sulfur (Fe-S) cluster proteins. This results in proteotoxic stress and, ultimately, cell death [18]. These observations indicate that copper has substantial potential for natural anti-apoptotic cancer therapy in tumor treatment. Several cuproptosis-related genes (CRGs) have been identified [18]. Therefore, studying the molecular biological characteristics of CRGs would help elucidate the reasons behind the heterogeneity of PTC and could provide new approaches for prognosis and treatment.

To evaluate the association of CRGs and the clinical characteristics of PTC patients, we constructed a prognostic cuproptosis signature based on CRG gene set variation analysis (GSVA) scores in this study [19]. This signature has a significant value in predicting the prognosis and the immune microenvironment of PTC, which will offer new insights into precisely identifying high-risk PTC patients and tailoring personalized treatments accordingly.

## 2 MATERIALS AND METHODS

### 2.1 Data Download and Preprocessing

Expression profile data (FPKM), genomic data (SNV and CNV), and clinical data of PTC were downloaded from The Cancer Genome Atlas (TCGA) using the R package TCGAbiolinks v1.16.0. To compare PTC and normal thyroid tissue, transcriptome data (FPKM) processed by the Toil RNA-seq Recompute pipeline were downloaded from UCSC Xena. Survival data used in this study were collected in 2018 [20]. Meanwhile, genes that were not expressed in more than 50% of the samples were removed.

### 2.2 Differential Analysis

Differences in gene expression were calculated using the Wilcoxon rank sum test. Genes with an absolute value of log2(Fold change) >1 and a corrected *P <* 0.05 were selected as differentially expressed genes. Multiple correction tests were analysed using the BH method. The expression heatmap of CRGs under different clinical features in PTC was drawn using the R package pheatmap (1.0.12).

### 2.3 Correlation Analysis

Correlation analysis was performed on the expression of CRGs in the PTC of the TCGA database, and a correlation heatmap was drawn using pheatmap (1.0.12).

### 2.4 Functional Enrichment Analysis

The Gene Ontology (GO) and Kyoto Encyclopedia of Genes and Genomes (KEGG) pathway enrichment analyses of significantly differentially expressed genes were performed utilizing the R package clusterProfiler (4.2.2). The results with an FDR-corrected p-value less than 0.05 were selected. The GSEA method was utilized to carry out further enrichment of KEGG pathways, and only those pathways with the FDR-corrected p-value below 0.05 were selected.

### 2.5 Comparison of CRGs Under Different Clinical Features

Based on the FPKM expression data and clinical characteristic data of PTC in TCGA, the differences in the expression of CRGs between different groups (such as age, gender, and stage) were calculated by the Wilcoxon rank sum test. The box diagram was created using ggpubr (0.4.0), and the heatmap was drawn using ComplexHeatmap (2.10.0).

### 2.6 Building a Protein-Protein Interaction Network

A protein-protein interaction (PPI) network was constructed using STRING (https://www.string-db.org/) based on the 10 CRGs [18], and functional enrichment analysis was also performed.

### 2.7 Construction of Cuproptosis-related Gene Signature and Survival Analysis

CRG scores were calculated based on the GSVA method [19]. Then, high and low cuproptosis score groups were analyzed based on the calculated median CRG score. Differentially expressed genes were identified between the two groups. Univariate Cox regression with a P-value of < 0.05 was performed to screen prognosis-related genes among the differentially expressed genes. Then, a CRG signature was constructed by performing the least absolute shrinkage and selection operator (LASSO) Cox regression. The risk score was calculated according to the risk formula previously reported [21-23]:


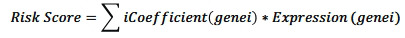


Kaplan-Meier curves were constructed using the R package survival (3.3-1), and the log-rank test was used to assess the effect on prognosis. The receiver operator characteristic (ROC) curve was performed using the R package timeROC (0.4). Cox regression (R package survival, 3.3-1) was used to calculate hazard ratios (HR) for score grouping and clinical characteristics.

### 2.8 Gene Mutation and Copy Number Variation Analyses

Based on single nucleotide mutation and copy number variation data of PTC in TCGA, the R package ComplexHeatmap (2.10.0) and oncoPrint function were used to display the top 30 genes in the two groups with high and low mutation frequencies, respectively. The R package GenVisR (1.26.0) was used to show the copy number variation of the low and high groups, which defines a copy number < 1 as a low copy number variation and a copy number > 3 as a high fold variation.

### 2.9 Immune Cell Infiltration Calculation

Based on the ESTIMATE, MCP-counter, XCELL, and CIBORSORT immune cell infiltration algorithms, the scores of each immune cell in the PTC samples were calculated using the R package IBOR (0.99.9). The Wilcoxon rank sum test was used to compare immune infiltration between the high and low cuproptosis score groups.

### 2.10 Tissue Samples and Quantitative Real-time PCR

Total RNA was extracted from 60 pairs of PTC and adjacent normal tissues from patients at Zhejiang Cancer Hospital. The study was approved by the Ethics Committee of Zhejiang Cancer Hospital. The RNA was obtained by using a TRIzol reagent (Beyotime, Shanghai, China). The purity and concentration of the extracted RNA were measured using nanodrop2000. Then, PrimeScript RT Master Mix (Takara, Dalian, China) was purchased to obtain cDNA. After that, the quantitative real-time polymerase chain reaction (qRT-PCR) was performed with SYBR Premix Ex Taq II (Takara, Dalian, China) on the LightCycler 480 system (Roche, USA). The housekeeping gene GAPDH was selected as an endogenous control. The primers were synthesized by Sangon Biotech (Shanghai, China), and the sequences were as follows: CDKN2A 5’- GGGTTTTCGTGGTTC ACATCC-3’ (forward) and 5’- CTAGACGCTGGCTCC TCAGTA-3’ (reverse), and GAPDH 5’-CAGGAGGCATTG CTGATGAT-3’ (forward) and 5’-GAAGGCTGGGGC TCATTT-3’ (reverse). The relative RNA expression was quantified with the 2−ΔΔCt method (Supplementary material - clinical data of 60 PTC patients).

### 2.11 Statistical Analyses

All statistical analyses of this study were performed using R software, version 4.1.0. *P <* 0.05 was considered statistically significant.

## 3 RESULTS

### 3.1 CRGs are Differentially Expressed in PTC

**Fig. (1):**
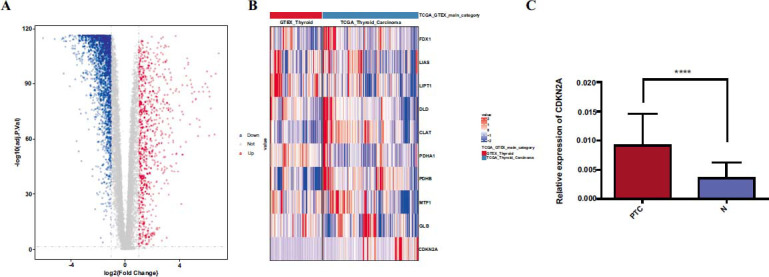
The differences in the expression of cuproptosis-related genes (CRGs) in PTC and normal thyroid tissues. (**A**) Volcano plot of differentially expressed genes (blue represents down-regulated genes in PTC, red represents up-regulated genes in PTC, and gray represents insignificant genes) using TCGA PTC sample and GTEX normal sample data for differential analysis. (**B**) The expression of 10 CRGs in PTC and normal thyroid tissues in TCGA PTC sample and GTEX normal sample data. (**C**) Validation of CDKN2A expression in 60 pairs of PTC and adjacent normal tissues detected by qRT-PCR. ****p< 0.0001.

On the basis of the TCGA-TARGET-GTEX FPKM dataset from UCSC Xena, we conducted a differential analysis between PTC and normal thyroid tissues. The analysis revealed the presence of 3,206 differentially expressed genes, with 2,340 being downregulated and 866 upregulated (Fig. **1A**). The gene expression (Z-Score) was scaled, and a heat map (Fig. **1B**) was drawn with a scale function of R. Among these differentially expressed genes, we found that the CRG of CDKN2A has an up-regulated trend in PTC (log2(Fold change) = 2.12, p = 6.48×10E-35), which was validated by qRT-PCR in 60 pairs of PTC and adjacent normal tissues (Fig. **1C**). The baseline characteristics of these 60 pairs of PTC patients showed that patients in the high CDKN2A expression group were more likely to have larger tumors than patients in the low CDKN2A expression group (Supplementary Table **S1**). This suggests that there might be a significant relationship between CDKN2A expression levels and clinical characteristics of PTC patients, and the CRGs may play a certain part in the tumorigenesis of PTC.

### 3.2 CRGs are Differentially Expressed in Different Clinical Features

**Fig. (2):**
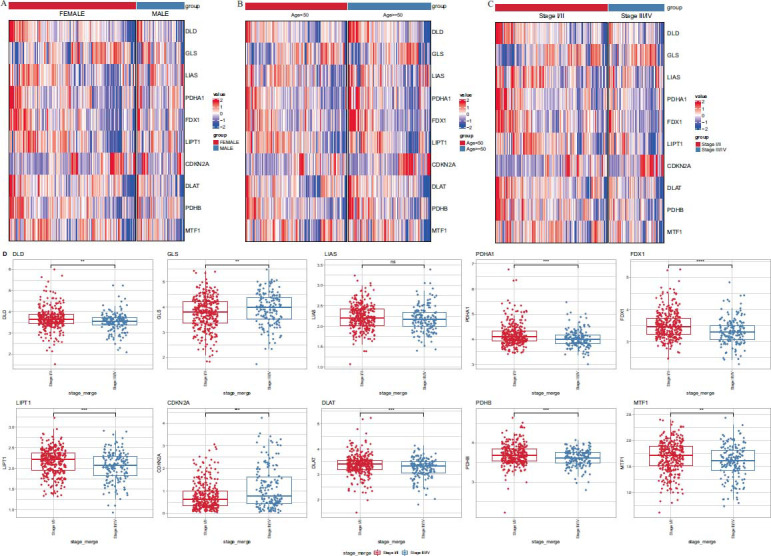
(**A, B, C**) Heat map of the expression of CRGs under different age, gender, and stage groups. (**D**) The differential analysis of CRGs between stage groups, **p< 0.01, ***p< 0.001, ****p< 0.0001, ns, not significant.

Based on the TCGA PTC data, we compared the differences in the expression of 10 CRGs between different clinical feature groups [18]. We observed that CRGs exhibited distinct expressions across various characteristics, such as gender, age, and stage. Significant differences were found in the expression of CRGs (Figs. **2A**-**C**). Especially among the staging groups, nine genes (FDX1, LIPT1, DLD, DLAT, PDHA1, PDHB, MTF1, GLS, and CDKN2A) were significantly differently expressed except for LIAS (Fig. **2D**), indicating that CRGs may play an important role in the development of PTC.

### 3.3 CRG Protein-Protein Interaction (PPI) Network

**Fig. (3):**
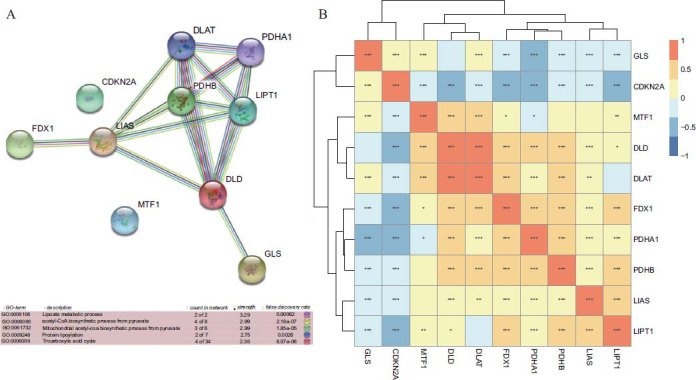
CRG PPI network and expression correlation. (**A**) PPI network and partial results of GO enrichment of 10 CRGs. (**B**) Heat map of expression correlation of 10 CRGs in PTC.

We constructed a PPI network based on the 10 CRGs (Fig. **3A**). After GO enrichment analysis, the implicated genes were found to be significantly enriched in multiple pathways, such as compound biosynthesis and energy metabolism. Furthermore, by Pearson correlation analysis of gene expressions, we detected that the expression patterns of PDHA1, LIAS, LIPT1, DLD, DLAT, FDX1, and PDHB were positively correlated, whereas CDKN2A was negatively correlated with the expression of multiple genes (Fig. **3B**).

### 3.4 Construction of a CRG Prognostic Signature

**Fig. (4):**
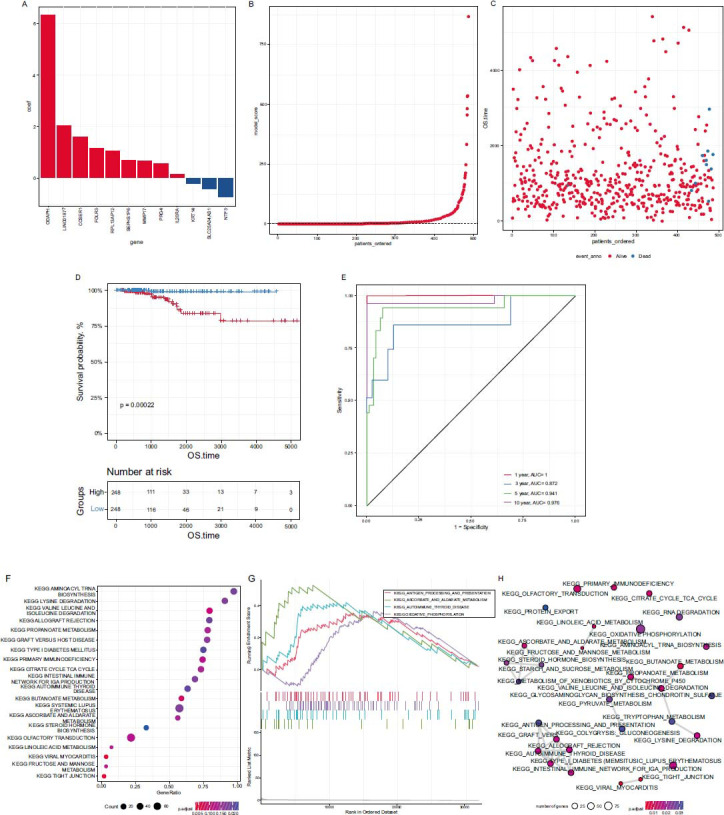
A CRG prognostic signature. (**A**) Model characteristic coefficients. (**B**) Patients are ranked based on CRG scores. (**C**) Survival status and time distribution of patients. (**D**) Survival analysis of patients grouped by high and low CRG scores. (**E**) Time-dependent 1-year, 3-year, 5-year, and 8-year ROC curves for CRG scores. (**F**, **G**, **H**) GSEA enrichment pathway.

Based on the CRG GSVA score grouping, gene expression differences were calculated, and these differentially expressed genes were screened for prognosis-related genes. After removing redundant genes, a signature was constructed based on the prognosis-related genes (Figs. **4A**-**C**). The samples were divided into two groups based on the median CRG score, and the results showed that it could effectively distinguish the overall survival (OS) of patients (Fig. **4D**), performing well in predicting patients' survival at 3 years, 5 years, and 8 years (AUC value 0.872, 0.941, and 0.976, respectively; Fig. **4E**). The differential expressed genes of the two groups with high and low CRG scores were calculated based on the grouping of CRG scores. Then, GSEA enrichment analysis was carried out for these differentially expressed genes, and some pathways were enriched, such as antigen presentation and energy metabolism (Fig. **4F**). The GSEA diagram of the enriched pathways is displayed in (Figs. **4G** and **H**).

**Fig. (5):**
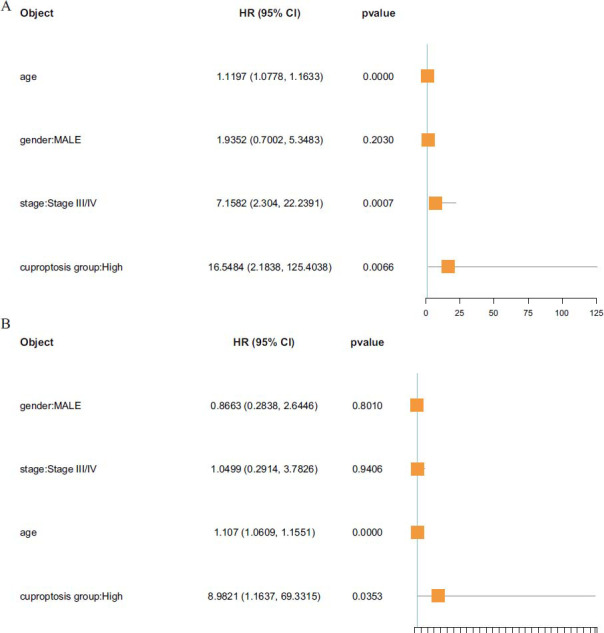
(**A**) Univariate Cox analysis grouped by gender, age, stage, and CRG scores. (**B**) Multivariate Cox analysis grouped by gender, age, stage, and CRG scores.

Incorporating gender, age, stage, and CRG score grouping into univariate and multivariate Cox regression analysis, it was found that CRG score grouping can be used as an independent prognostic factor (Univariate: HR = 16.5484, 95% CI = 2.1838 – 125.4038, *p* = 0.0066; multivariate: HR = 8.9821, 95% CI = 1.1637– 69.3315, *p* = 0.0353; Figs. **5A** and **B**).

**Fig. (6):**
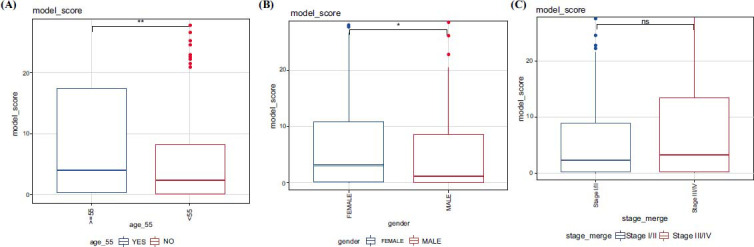
Differential analysis of CRG scores among clinical features. Differential analysis for (**A**) age, (**B**) gender, and (**C**) stage using the Wilcox test (ns: *P >* 0.05, *: *P <* 0.05, **: *P <* 0.01).

We further analyzed the TCGA dataset and found that there were differences in CRG scores in age (*P <* 0.01) and gender groups (*P <* 0.05; Figs. **6A** and **B**), but no differences in CRG scores were observed in stage groups (Fig. **6C**).

### 3.5 Molecular Features and Pathway Analysis of CRG Signature

**Fig. (7):**
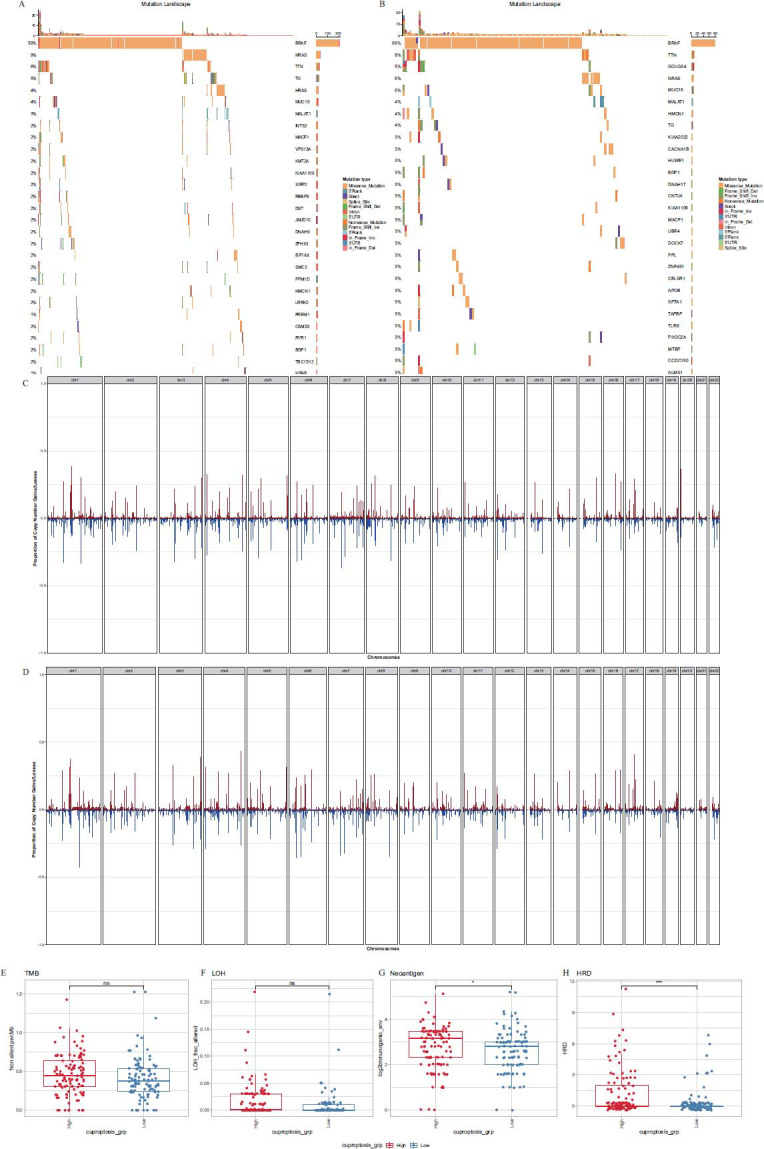
Relationship between CRG signature and single nucleotide mutation and copy number variation. (**A, B**) Mutation waterfall diagram of the top 20 genes in the high CRG score group (HCSG, **A**) and low CRG score group (LCSG, **B**). (**C, D**) Autosomal copy number variation in the HCSG (**C**) and LCSG (**D**). (**E, F, G, H**) Differences of tumor mutation burden (TMB, **E**), loss of heterozygosity (LOH, **F**), neoantigen (**G**), and homologous recombination deficiency (HRD, **H**) in the HCSG and LCSG (ns: *P >* 0.05, *: *P <* 0.05, ***: *P <* 0.001).

Based on TCGA single nucleotide mutation and copy number variation data of PTC, the CRG signature was divided into two groups according to the median CRG score, and the mutations of the top 20 genes in both high CRG score group (HCSG) and low CRG score group (LCSG) are shown in Figs. (**7A** and **B**), respectively. We found that both HCSG and LCSG contained high-frequency mutations of the BRAF gene, but there were no significant differences in gene mutations between the two groups. In terms of copy number variation, we characterized the amplifications and deletions of copy number on 22 autosomes in the HCSG and LCSG and found that HCSG had more copy number amplifications and deletions on chromosomes 7 and 12 (Figs. **7C** and **D**). Then, we compared the differences in other genomic alterations, such as tumor mutation burden (TMB), loss of heterozygosity (LOH), neoantigen, and homologous recombination deficiency (HRD) between the HCSG and LCSG, and found that there were more neoantigen and HRD in HCSG than in LCSG while there was no significant difference of TMB and LOH in two groups (Figs. **7E**-**H**).

**Fig. (8):**
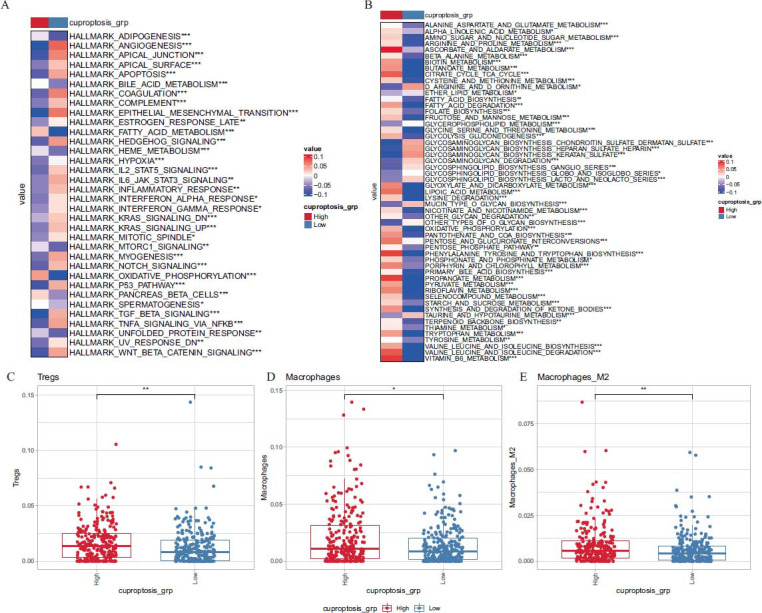
(**A**) Heatmap of differences of Hallmark pathway enrichment scores between HCSG and LCSG. (**B**) Heat map of the differences of KEGG metabolic pathway enrichment scores between HCSG and LCSG. (**C**, **D**, **E**) Proportions of (**C**) Tregs, (**D**) Macrophages, and (**E**) Macrophages M2 between HCSG and LCSG. (*: *P <* 0.05, **: *P <* 0.01, ***: *P <* 0.001).

The tumor hallmark pathway and KEGG metabolic pathway enrichment scores were calculated based on GSVA, and the differences in the hallmark and KEGG metabolic pathways between HCSG and LCSG were evaluated. The mean values of pathway scores between HCSG and LCSG were calculated and presented using heat maps. It was observed that there were significant differences in hallmark pathways and KEGG metabolic pathways between HCSG and LCSG, respectively (Figs. **8A** and **B**).

Immune cell infiltration was also calculated between HCSG and LCSG. It was found that the immune suppression cell score in HCSG was significantly higher than that in LCSG. Meanwhile, correlation analysis was conducted on the proportion of immune cells and CRG scores, but only a few cells were significantly correlated with CRG scores (Figs. **8C**-**E**).

## 4 DISCUSSION

Recent studies have identified cuproptosis, which refers to the fact that excess intracellular copper can be transported to mitochondria *via* ion carriers and bind directly to lipoylated components of the TCA cycle, leading to the aggregation of lipoylated proteins and the loss of Fe-S cluster proteins, thus resulting in proteotoxic stress and ultimately cell death [18]. Copper homeostasis has increasingly been recognized as a factor influencing tumor growth and triggering tumor cell death [24]. Copper plays an important role in tumor immunity and antitumor therapy [25-27]. Here, we established a CRG signature and evaluated the prognostic value in PTC. In addition, we explored the role of CRGs in the development of tumor microenvironment (TME) and its potential therapeutic value in PTC.

In this study, we explored the expression feature of 10 CRGs (FDX1, LIPT1, DLD, LIAS, DLAT, PDHA1, PDHB, MTF1, GLS, and CDKN2A) in PTC [18]. It was found that CDKN2A was differentially expressed between tumor tissues and normal tissues, and there was an upregulated trend in PTC, which was verified by qRT-PCR. In addition, we also detected that all CRGs, except for LIAS, were significantly differentially expressed in different stages, in which GLS and CDKN2A were positively correlated with stage. GLS is regulated by oncogenes and largely promotes the growth of tumor cells [28]. Previous studies indicated that CDKN2A/p16 is a known tumor suppressor gene [29]. Decreased CDKN2A expression is a marker of poor prognosis in high-grade gliomas, while poor prognosis in ovarian and bladder cancers is associated with increased CDKN2A expression [30]. At least three silencing modalities of CDKN2A (pure deletion, promoter methylation, and point mutations) are associated with poorer clinical outcomes in some cancers [31]. In meningiomas, elevated CDKN2A mRNA expression may serve as a biomarker of clinical aggressiveness, and deletion of pure-transgenic CDKN2A/B will result in a worse prognosis [32]. In PTC, although our statistical results only indicate the association between CDKN2A and tumor size due to insufficient sample size, there is evidence from previous studies suggesting that CDKN2A plays a crucial role in promoting tumor cell proliferation and invasion [33, 34]. We will also increase the sample size in the future to validate these findings further. All these indicate that cuproptosis may be related to PTC tumorigenesis or progression. Next, to explore the biological functions of these 10 CRGs, we performed enrichment analysis and found that these CRGs were enriched in the TCA cycle, glycolysis, and fatty acid metabolic pathways, which was consistent with the conclusion that copper induces cell death by targeting the lipoylated TCA cycle. Notably, the relationship between CRGs and PTC needs further elaboration.

Subsequently, we screened 12 prognostic genes based on CRG scores to construct a CRG prognostic signature. Our findings indicated that the CRG score emerged as an independent prognostic indicator for predicting survival outcomes in PTC patients after controlling for confounders. The ROC curve confirmed its good predictive validity for 3-year, 5-year, and 8-year OS. In conclusion, the CRG score may have great predictive power for the prognosis of PTC patients. We divided the signature into HCSG and LCSG based on CRG scores. Both groups contained high-frequency mutations in the BRAF gene, which is the most common genetic alteration in PTC [26, 35, 36]. Therefore, CRGs are critical to the study of PTC. Then, we analyzed the copy number variation data and found that more copy number amplifications and deletions occurred in chromosomes 7 and 12 in the HCSG, and we speculated whether cuproptosis could be influenced by genes on these chromosomes, which warrants further investigation.

Immune response holds pivotal importance in the development of tumors, highlighting its potential significance as a target in cancer therapy. For the differential genes of HCSG and LCSG, we performed functional enrichment analysis. The results showed that these differential genes were enriched in functions, such as antigen presentation and energy metabolism, suggesting that CRGs may be associated with immune responses. Neoantigens are self-antigens produced by tumor cells as a result of genomic mutations. Neoantigens have the distinct advantages of unique tumor specificity and absence in normal tissues, providing ideal targets for effective individualized tumor therapy [37]. In our study, we found significant mutational differences between HCSG and LCSG, with significantly higher neoantigen and HRD scores in HCSG. This suggests that immunotherapy may be more effective in HCSG and provides a new idea for immunotherapy of tumors [38].

Tumors typically exhibit distinct gene expression patterns at both the mRNA and protein levels. In recent years, it has been observed that the TME also has significant heterogeneity, and its role in tumor development has garnered increasing attention [39-41]. In TME, immune cells and stromal cells are identified as the major components [42]. Tumor-associated immune cells may have either tumor-antagonistic or tumor-promoting functions [43]. The cells that promote tumor-promoting function include regulatory T cells (Tregs) and myeloid-derived suppressor cells (MDSCs). Treg, as an immunosuppressive type of cell, is a double-edged sword. On one hand, Treg functions to suppress overreactive immune responses, particularly in autoimmune diseases. On the other hand, their inhibitory activity within the TME could potentially hinder the effectiveness of cytotoxic T cells (CTLs) in targeting cancer cells [44]. Previous single-cell sequencing results reported that Tregs exhibit high heterogeneity in tumors, and the function of Tregs is strongly correlated with patient prognosis [45-47]. Tumor-associated M2-polarized macrophages have been considered as the main source of MDSCs [48]. MDSCs can promote angiogenesis by producing MMP9, prokineticin 2, and vascular endothelial growth factor (VEGF). Additionally, MDSCs facilitate the migration of cancer cells toward the endothelium, thereby enhancing the metastasis of tumors [49, 50]. In this study, we found that, based on certain immune cell infiltration algorithms, the score for immunosuppressive type cells in HCSG was significantly higher than that in LCSG. This finding aligns with the conclusion that HCSG has a worse prognosis and also suggests that CRGs may be involved in modulating the TME, especially immunosuppressive cells, thus promoting tumor growth and progression. Therefore, targeting CRGs may be a new strategy for effective treatment of PTC.

## CONCLUSION

This study evaluated the expressions of CRGs and their relationships with clinical features in PTC patients. Based on the CRG prognostic signature, PTC was divided into two subtypes (HCSG and LCSG), which showed significant differences in prognosis and immune microenvironment. The results of the study may be used by clinicians to make treatment plans for PTC patients, especially for the evaluation of immunotherapy. This study also provides an important basis for further research on the role of cuproptosis in PTC.

## STUDY LIMITATIONS

Our study has several limitations. Although the differential expression of CDKN2A was validated through bioinformatics analysis and qRT-PCR, further *in vitro* or *in vivo* experiments are needed to elucidate the molecular mechanisms by which CRGs contribute to PTC progression. Furthermore, prospective studies are necessary to assess the practical utility of the CRG signature.

## Data Availability

The data and supportive information are available within the article.

## LIST OF ABBREVIATIONS

AUC: Area Under the Curve
CRG: Cuproptosis-Related Gene
CTLs: Cytotoxic T Cells
DNA: Deoxyribonucleic Acid
Fe-S: Iron-Sulfur
GO: Gene Ontology
GSVA: Gene Set Variation Analysis
HCSG: High CRG Score Group
HR: Hazard Ratios
HRD: Homologous Recombination Deficiency
KEGG: Kyoto Encyclopedia of Genes and Genomes
LASSO: Least Absolute Shrinkage and Selection Operator
LCSG: Low CRG Score Group
LOH: Loss of Heterozygosity
MDSCs: Myeloid-Derived Suppressor Cells
OS: Overall Survival
PPI: Protein-Protein Interaction
PTC: Papillary Thyroid Cancer
qRT-PCR: Quantitative Real-time Polymerase Chain Reaction
T4: Thyroid Hormones
TC: Thyroid Cancer
TCA cycle: Tricarboxylic Acid Cycle
TCGA: The Cancer Genome Atlas
TMB: Tumor Mutation Burden
TME: Tumor Microenvironment
Tregs: Regulatory T Cells
VEGF: Vascular Endothelial Growth Factor
