# Supplementary material for: A Novel Cuproptosis-Related Gene Signature Predicts Prognosis in Papillary Thyroid Carcinoma Patients
Source: Comb Chem High Throughput Screen. 2024 Aug 27;28(14):2463–76. doi: 10.2174/0113862073333880240819105537 (PMC12728525; doi:10.2174/0113862073333880240819105537)
Supplement: Supplementary file 1 [file CCHTS-28-14-2463_SD1.pdf]

Supplementary Material

A Novel Cuproptosis-Related Gene Signature Predicts Prognosis in Papillary Thyroid Carcinoma Patients

Jun Cao[1#], Shijia Zhang[2,3,#], Kehui Zhou[2,3], Xiaochun Mao[3,4], Ming Zhao[3,4], Jinbiao Shang[3,4] and Xiabin Lan[2,3,4,*]

[1]*Department of Head and Neck and Rare Oncology, Zhejiang Cancer Hospital, Hangzhou Institute of Medicine (HIM), Chinese Academy of Sciences, Hangzhou, Zhejiang 310022, China;* [2]*Postgraduate Training Base Alliance of Wenzhou Medical University (Zhejiang Cancer Hospital), Hangzhou, Zhejiang, 310022, China;* [3]*Department of Thyroid Surgery, Zhejiang Cancer Hospital, Hangzhou Institute of Medicine (HIM), Chinese Academy of Sciences, Hangzhou, Zhejiang 310022, China;* [4]*Key Laboratory of Head & Neck Cancer Translational Research of Zhejiang Province, Hangzhou, Zhejiang 310022, China*

Baseline characteristics of 60 patients with papillary thyroid carcinoma

Parameters		CDKN2A low expression（n=30）	CDKN2A high expression（n=30）	p-Value
**Age (year)**				
	＜55	25（83.3%）	24（80.0%）	0.739
	⩾55	5（16.7%）	6（20.0%）	
**Gender**				
	Male	10（33.3%）	11（36.7%）	0.787
	Female	20（66.7%）	19（63.3%）	
**Size（cm）**				
	＜3	30（100%）	24（80.0%）	**0.010**
	⩾3	0（0%）	6（20.0%）	
**PTMC**				
	Yes	7（23.3%）	2（6.7%）	0.071
	No	23（76.7%）	28（93.3%）	
**Multifocal cancer**				
	Yes	14（46.7%）	21（70.0%）	0.067
	No	16（53.5%）	9（30.0%）	
**Extrathyroidal extension**				
	Yes	20（66.7%）	17（56.7%）	0.426
	No	10（33.3%）	13（43.3%）	
**Lymph node metastasis**				
	Yes	22（73.3%）	24（80.0%）	0.542
	No	8（26.7%）	6（20.0%）	
**T grade**				
	T1+T2	20（66.7%）	15（50.0%）	0.190
	T3+T4	10（33.3%）	15（50.0%）	
**N grade**				
	N0+N1a	14（46.7%）	17（56.7%）	0.438
	N1b	16（53.3%）	13（43.3%）	
**Stage**				
	Ⅰ-Ⅱ	20（66.7%）	17（56.7%）	0.426
	Ⅲ-Ⅳ	10（33.3%）	13（43.3%）	
